# Radiogenomics predicts the expression of microRNA-1246 in the serum of esophageal cancer patients

**DOI:** 10.1038/s41598-020-59500-7

**Published:** 2020-02-13

**Authors:** Isamu Hoshino, Hajime Yokota, Fumitaka Ishige, Yosuke Iwatate, Nobuyoshi Takeshita, Hiroki Nagase, Takashi Uno, Hisahiro Matsubara

**Affiliations:** 1 Division of Gastroenterological Surgery, Chiba Cancer Center, Chiba, Japan; 2 Department of Diagnostic Radiology and Radiation Oncology, Graduate School of Medicine, Chiba University, Chiba, Japan; 3 Department of Hepatobiliary and Pancreatic Surgery, Chiba Cancer Center, Chiba, Japan; 4 Division of Surgical Technology, National Cancer Center Hospital East, Kashiwa, Japan; 5 Laboratory of Cancer Genetics, Chiba Cancer Center Research Institute, Chiba, Japan; 6 Department of Frontier Surgery, Graduate School of Medicine, Chiba University, Chiba, Japan

**Keywords:** Oesophageal cancer, Tumour biomarkers, Cancer imaging

## Abstract

Radiogenomics is a new field that provides clinically useful prognostic predictions by linking molecular characteristics such as the genetic aberrations of malignant tumors with medical images. The abnormal expression of serum microRNA-1246 (miR-1246) has been reported as a prognostic factor of esophageal squamous cell carcinoma (ESCC). To evaluate the power of the miR-1246 level predicted with radiogenomics techniques as a predictor of the prognosis of ESCC patients. The real miR-1246 expression (miR-1246_real_) was measured in 92 ESCC patients. Forty-five image features (IFs) were extracted from tumor regions on contrast-enhanced computed tomography. A prediction model for miR-1246_real_ was constructed using linear regression with selected features identified in a correlation analysis of miR-1246_real_ and each IF. A threshold to divide the patients into two groups was defined according to a receiver operating characteristic analysis for miR-1246_real_. Survival analyses were performed between two groups. Six IFs were correlated with miR-1246_real_ and were included in the prediction model. The survival curves of high and low groups of miR-1246_real_ and miR-1246_pred_ showed significant differences (p = 0.001 and 0.016). Both miR-1246_real_ and miR-1246_pred_ were independent predictors of overall survival (p = 0.030 and 0.035). miR-1246_pred_ produced by radiogenomics had similar power to miR-1246_real_ for predicting the prognosis of ESCC.

## Introduction

According to the World Health Organization report, the number of patients with esophageal squamous cell carcinoma (ESCC) is on an increasing trend; it is considered to be the sixth most common types of cancer in men and the thirteenth most common types of cancer in women^1^. Although treatment for ESCC has been improved in recent years, the prognosis is still quite poor, according to the report the 5-year survival rate is less than 30%^2,3^. Now, more than ever, technological developments such as the prediction of the diagnosis and treatment effects are desired to improve the management of ESCC. On January 20^th^, 2015, Barack Obama, the former President of the United States of America, announced the “Precision Medicine Initiative” in the State of the Union address, attracting worldwide attention^4^. In precision medicine, patients are analyzed and the optimal therapy is selected at an individual level. However, additional examinations and treatments for each patient can directly raise medical expenses. In 2018, it cost more than $1000 to perform a genetic analysis and the cost-effectiveness remains controversial^5^.

In contrast, medical imaging modalities, such as computed tomography (CT) and magnetic resonance imaging are widely used in the diagnosis of diseases and symptoms, including malignant tumors because the features of tissue images are strongly correlated with histopathological findings^6,7^. In this century, new correlations are being identified between tissue scale imaging and cellular molecule data based on the accumulation of comprehensive genome and transcriptome data from gene expression analyses using next generation sequencing and other approaches^8–11^. Radiogenomics is a method of integrating and analyzing the results of radiomics (the analysis of radiological imaging) and omics analyses (*e*.*g*., genome and gene expression analyses (genomics))^12,13^. Therefore, image features (IFs) can be used as non-invasive biomarkers. In other words, it is possible to use IFs and omics information such as genome and epigenome that correlate with clinical features, and as a result, to predict prognosis, diagnosis, and treatment response from imaging^14^. The actual IFs are extracted from the segmented regions, and these features can be broadly classified into statistics, shapes, textures, morphology, motion, and enhancements^15^. Positive and/or negative associations between IFs and omics features such as genomes and epigenomes are examined using statistical methods. Second, predictive models can be built using a number of statistical and deep machine learning algorithms^16^. Marigliano C. *et al*. extracted RNA from tissue samples of 20 cases of clear cell renal cell carcinoma and performed microRNA analysis to identify five candidate microRNAs (miRs) that expressed differently from normal tissues^17^. At the same time, Radiogenomics analysis was performed using CT images of the same patient, and it was confirmed that there was a correlation between the expression of these five miRs and image features. They also showed that, in particular, miR-21-5p, which is involved in tumor progression, has a strong correlation with one of the image features, entropy.

MiRs are non-coding single stranded RNAs that are characterized by their size (approximately 17–25 nucleotides) and are transcribed from the genome^18–20^. Although their function has not been clear, in recent years it has been demonstrated that miRs are combined with mRNA with a complementary sequence, and that they inhibit targets by translation inhibition and cleavage (degradation)^21–23^. To date, more than 1000 miRs have been identified in humans^24^. It is now becoming clear that these miRs are aberrantly expressed in a disease-specific manner, specific to the cancer type, and that miRs circulating in the blood are relatively stable^25–28^. We previously reported that miR-1246 is significantly expressed in the serum of esophageal cancer patients and that its expression is an independent predictor of the prognosis^29^. Thus, miR is considered useful as a novel biomarker. Since analysis of miR expression requires time and economic cost, it is considered useful to apply general image examination to estimate its expression. In the present study, we attempted to predict the serum expression level of miR-1246 using radiogenomics techniques and to evaluate the power of the predicted miR-1246 level as a predictor of the prognosis of ESCC patients.

## Results

### miR-1246 is upregulated in ESCC serum samples

The serum miR-1246 expression levels of 35 healthy controls and all ESCC patients were evaluated by a qRT-PCR. The miR-1246 expression levels in serum from ESCC patients were significantly higher than those from controls (P < 0.001). The ROC analysis revealed that at the optimal cut off value of 1.3234 for miR-1246, the sensitivity was 71.29% and the specificity was 73.91%, with an area under the curve of 0.754.

### The analysis of the real miR-1246 expression

**Figure 1:**
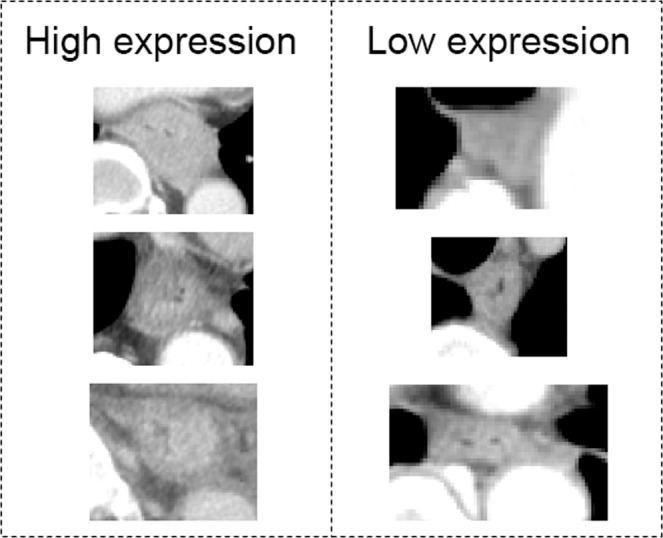
Examples of the high and low expression of serum miR-1246. The contrast uptake and wall thickness of the tumors in the high expression group appear much more evident in comparison to the low expression group.

A value of 15.0 was selected as the threshold for the miR1246 expression to divide the patients into two groups according to the ROC analysis of survivors and non-survivors. Examples of CT images in the miR-1246_real_-high and miR-1246_real_-low groups are shown in Fig. 1.

### Radiogenomics

**Figure 2:**
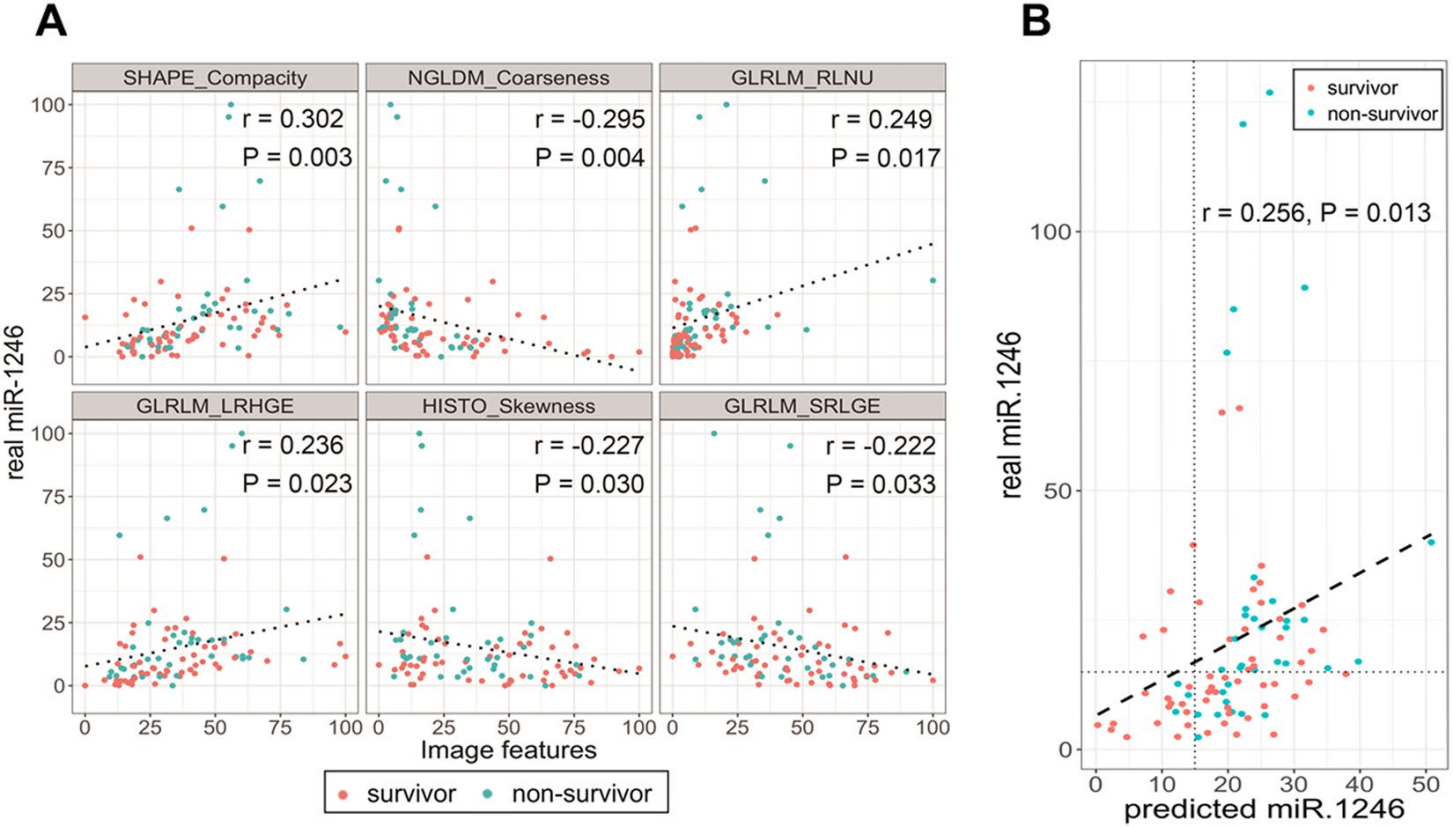
(**A**) Scatter plots between the serum miR-1246 expression and image features that were significantly correlated with the serum miR-1246 expression. Survivors and non-survivors are shown as red and blue points, respectively. (**B**) A scatter plot between the real and predicted miR-1246 values. The predicted miR-1246 value was derived from 6 image features that were identified in the correlation analysis. Survivors and non-survivors are shown as red and blue points, respectively. A significant correlation was identified when all data were used (r = 0.256; P = 0.013). Patients with miR-1246 expression values of more than 50 appeared to be outliers from the approximately straight line (dashed line), and 5 of the 7 patients with such values were non-survivors. When removing these data, the correlation was clearer (r = 0.452; P < 0.001).

SHAPE_Compacity, NGLDM_Coarseness, GLRLM_RLNU, GLRLM_LRHGE, HISTO_Skewness and GLRLM_SRLGE were significantly correlated with the miR-1246_real_ value (r = 0.301, −0.295, 0.249, 0.236, −0.227 and −0.222; P = 0.003, 0.004, 0.017, 0.023, 0.030 and 0.033, respectively) (Fig. 2A). The miR-1246_pred_ value calculated from linear regression with 10-fold cross-validation was significantly correlated with the miR-1246_real_ value (r = 0.256, P = 0.013) (Fig. 2B).

**Figure 3:**
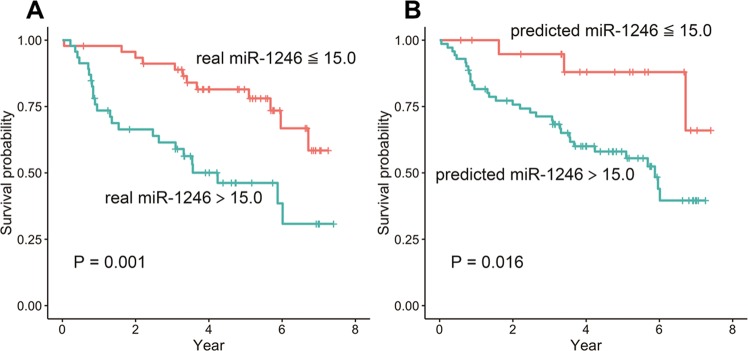
The survival curves of the real (**A**) and predicted (**B**) miR-1246 expression levels for values that were above or below the threshold (=15.0). A log rank test revealed significant differences between the curves respectively (P = 0.001 and 0.016).

A value of 15.0 was used as the threshold to divide patients into miR-1246_real_ and miR-1246_pred_ groups. Significant differences were observed between the miR-1246_real_-high and miR-1246_real_-low groups and between the miR-1246_pred_-high and miR-1246_pred_-low groups (P = 0.001 and 0.016, respectively) (Fig. 3A,B). The results of the Cox regression analyses are shown in Tables 1 and 2. In the univariate Cox regression analyses, N stage, miR-1246_real_ and miR-1246_pred_ showed statistical significance (Hazard ratio = 1.880, 1.018 and 1.068; P = 0.002, <0.001 and =0.003). According to a multivariate Cox regression analysis, only miR-1246_real_ was found to be significantly independent factors for N stage and miR-1246_real_ (Hazard ratio = 1.013; P = 0.030), whereas only miR-1246_pred_ was found to be a significantly independent factor for N stage and miR-1246_pred_ (Hazard ratio = 1.051; P = 0.035).

**Table 1:** The results of the univariate Cox regression analyses for survival.

Variable	Univariate	95% CI	P-value
	Hazard ratio		
Clinical factor			
age	1.006	0.964–1.049	0.792
sex	5.869	0.802–42.790	0.081
T stage	1.396	0.954–2.043	0.086
N stage	1.880	1.252–2.822	**0**.**002**
M stage	0.907	0.123–6.672	0.092
SCC	0.992	0.930–1.058	0.801
CEA	0.990	0.913–1.073	0.810
real miR.1246	1.018	1.008–1.028	**<0**.**001**
predicted miR.1246	1.068	1.023–1.114	**0**.**003**

^*^P values with statistical significance are written in bold. CI, confidence interval.

**Table 2:** The results of the multivariate variate Cox regression analyses for survival.

Variable	Hazard ratio	95% CI	P-value	Hazard ratio	95% CI	P-value
Clinical factor						
N stage	1.544	0.985–2.421	0.058	2.807	0.035–2.290	0.051
real miR.1246	1.013	1.001–1.025	**0**.**030**			
predicted miR.1246				1.051	1.004–1.101	**0**.**035**

^*^P values with statistical significance are written in bold. CI, confidence interval.

## Discussion

In this study, we examined the relationship between the expression levels of miR-1246 in serum and image features using Radiomics techniques. There seemed to be a close relationship between the miR-1246 expression and the image features. We first confirmed the abnormal expression of miR-1246 in ESCC patients in comparison to normal controls, which validated the findings of our previous report (Takeshita *et al*., 2013). Next, we set the VOI for the lesions on contrast-enhanced CT by referring to the FDG-PET and endoscopy findings, and extracted image features using the VOI. We then selected the image features that were significantly correlated with the serum level of miR-1246 using a correlation analysis. As a result, 6 image features (SHAPE_Compacity, NGLDM_Coarseness and so on) were extracted. The constructed model with the selected 6 features predicted serum levels of miR-1246. miR-1246_pred_ could divide the ESCC patients into two groups with a better and worse prognosis. Furthermore, the miR-1246_pred_ was an independent predictor of the prognosis. The patients were similarly divided by their miR-1246_real_ values; thus, for predicting the prognosis of ESCC patients, the predicted miR-1246 (determined on CT images) might be used as a substitute for the measurement of the miR-1246 expression by a qRT-PCR. Body fluid levels of miR-1246 have been reported to be good candidate biomarkers in various malignancies. Recently, a systematic review and meta-analysis revealed that miR-1246 had the high efficacy for discriminating gastrointestinal cancer patients from normal controls (sensitivity, 0.920; specificity, 0.958)^30^. Actually, serum miR-1246 was significantly elevated in ESCC patients, similar to our previous results^29^. However, no significant increase was observed in cStageI patients compared to healthy controls (P = 0.297, data not shown), which may be inappropriate for screening for early cancer. However, the value of cStageII patients was significantly higher than healthy controls (P = 0.0002), and the value also increased according to the degree of progression, suggesting that it may be an indicator of disease status.

Although the function of miR-1246 is still unclear, several reports have indicated the mechanisms of miR-1246. A number of miRs have been shown to play a role in p53-dependent growth control, apoptosis and senescence and miR-1246 is considered to be one of the targets of p53 protein since the promoter region has a p53-response element^31^. However, in order to clarify the relationship between p53 status and miR-1246 expression in esophageal squamous cell carcinoma and its action, more detailed examination is necessary.

Current evidence also suggests that miR-1246 can act as an oncogene. MiR-1246 promotes cancer stemness, including self-renewal, drug resistance, tumorigenicity, and metastasis, via the activation of the Wnt/β-catenin pathway in hepatocellular carcinoma. Clinically, high endogenous and circulating miR-1246 was identified in HCC clinical samples and was correlated with a worse prognosis^32^. Moreover, miR-1246 is considered a crucial driver for tumor initiation and the progression of cancer in human non-small cell lung cancer and the serum level of miR-1246 is correlated with the clinical response of lung cancer patients receiving anti-neoplastic therapy^33^. The miR-1246 expression was significantly upregulated in oral squamous cell carcinoma tumor tissues. The patient group with high miR-1246 expression levels had a worse survival rate in comparison to those with low expression levels. Besides, the inhibition of miR-1246 in oral cancer stem cells significantly reduced stemness hallmarks and the downregulation of miR-1246 decreased chemoresistance^34^. Thus, miR-1246 could be clinically useful both as a biomarker and therapeutic target.

Radiogenomics is a novel technology that allows for the non-invasive prediction of molecular characteristics of human malignancies^35^. Radiogenomics has been considered more efficient and effective than conventional imaging analyses, since the goal of this technology is to identify phenotypic imaging biomarkers that are related to the gene expression and/or mutations that provide predictive and prognostic information that can be used for the selection of personalized precision medicine^8,10,11^. In fact, in clinical cases, imaging examinations are always performed to diagnose the status of malignant tumors; thus, an alternative image marker that can analogize tumor-specific genetic changes would be very useful. Actually, several reports describing image features, focusing on the Ki-67 status of gliomas, have been published. Entropy, derived from apparent diffusion coefficient (ADC) maps of gliomas, was found to be associated with the expression of Ki-67^36^. Furthermore, in a small sample study (n = 21), features related to Ki-67 were identified among 86 radiological features extracted from conventional structural magnetic resonance (MR) images of glioblastoma^37^. Woodard GA. *et al*. showed that there was no significant difference in the risk of breast cancer recurrence in ER-positive breast cancer patients determined based on the amount of image features on Breast Imaging and Reporting Data System (BI-RADS) mammography and MR images and the risk determined based on an Oncotype DX assay^10^.

The correlation analysis identified 6 significantly correlated image features derived from 1 morphologic, 1 histogram and 4 texture analyses. The SHAPE_Compacity of a shape feature and NGLDM_Coarseness of a texture feature showed the strongest and second strongest correlations with the serum miR-1246 expression. SHAPE_Compacity reflects how compact the VOI is. As the VOI shape becomes spherical, compacity becomes small. In other words, if the esophageal cancer has progressed in the longitudinal and vertical directions, the compacity will be greater; eventually the miR-1246 level will also become high. NGLDM_Coarseness is defined as the level of the spatial rate of change in intensity. As the pixel values become more nonuniform, the value of NGLDM_Coarseness will decrease. Thus, when esophageal cancer progresses and the contrast uptake becomes heterogenous, miR-1246 will become high. Actually, when reviewing the images of ESCC, the contrast uptake and wall thickness of the tumors in the miR-1246-high group seemed to be more evident than that in the miR-1246-low group. These imaging findings would influence the NGLDM_Coarseness.

The correlation between miR-1246_real_ and miR-1246_pred_ was not high (r = 0.256). The result was affected by outlying miR-1246_real_ values. When these values were removed, the correlation value improved (r = 0.452). In order to interpret the results of machine learning easily and to avoid overfitting for the given data, we used a simple algorithm, linear regression, to construct the model in this study. In the next stage, non-linear machine learning models may be able to fit the outliers; however, appropriate manipulation and larger datasets are essential to avoid overfitting.

Our study was associated with several limitations. First, the study population was relatively small, which might have affected the stability of the predictive model. Although linear regression is useful for reducing the likelihood of overfitting and despite the fact that we performed cross-validation to evaluate the generalization ability of the model, further validation with data from an external institute would be useful. Second, the segmentation was manual. To prevent VOI delineation from affecting the results, a well-experienced radiologist and surgeon delineated the VOI with consensus and also referred to the FDG-PET and endoscopy images. Moreover, the VOI shape was simplified by covering the wall and lumen of the slice visualizing the tumor.

In conclusion, the present study suggested that the serum level of miR-1246 could be inferred from image features, and the findings are considered to provide a foothold for information analysis systems that predict molecular information from image information. It is considered that there have been no reports of analysis of marker expression in serum using the Radiogenomics. Moreover, the findings of the present study suggest that the image features of tumors with high miR-1246 expression levels might be associated with the tumor extension shape, and it is presumed that the tumor density presents heterogeneity. It is an important fact for the development of Radioepigenomics that even independent prognostic factors in serum such as miR1246 are reflected to some extent in image features and can be predicted from images. Based on this result, we are now planning to examine Radiogenomics technology for clinically useful markers such as, PD-L1, HER2, and BRACA1, etc. In the future, the multimodal so-called “RadioEpigenomics” approach, through which molecular information is predicted based on the analysis of radiological imaging, will be applied in precision medicine.

## Materials and Methods

### Patients

The protocol was approved by the Institutional Review Board of Chiba Cancer Center (No. 28–15) and all patients and healthy volunteers provided their written informed consent. The study was carried out in accordance with the World Medical Association’s Declaration of Helsinki. In total, 101 patients were diagnosed with ESCC in our hospital between October 2010 and July 2017. Ninety-two of one hundred one patients (age 65.0 ± 8.0 years (mean ± standard deviation); female, n = 12; male, n = 80) underwent contrast-enhanced CT before any therapeutic procedures, including surgery, chemotherapy and radiotherapy, and were followed up for more than 2 years. The demographics of the 92 ESCC patients are shown in Table 3.

**Table 3:** Patient details and clinicopathological features.

Characteristics	No. of Patients (%)
**Sex**	
Male	80 (87.0)
Female	12 (13.0)
**Age**	65.9 (41–87)
**pT category**	
T1a or b	28 (30.5)
T2	12 (13.0)
T3 or T4	52 (56.5)
**pN category**	
N0	49 (53.3)
N1	28 (30.5)
N2	13 (14.1)
N3	1 (1.1)
**M category**	
M0	5 (5.4)
M1	87 (94.6)
**Stage**	
I	26 (28.3)
II	24 (26.1)
III	35 (38.0)
IV	7 (7.6)
**SCC (ng/mL)**	2.0 ± 4.5
**CEA (mg/mL)**	4.1 ± 4.7

mean ± standard deviation.

### miR-1246 expression analysis

#### Collection of serum samples

Venous blood samples were collected from all ESCC patients. These samples were collected before any therapeutic procedures. Thirty-five healthy controls without any history of malignant disease were included in the test cohort. Samples were stored at −80 °C. Written consent for sample donation for research purposes was obtained from each patient before sample collection.

#### RNA extraction

Total RNA that contained small RNAs was extracted from 400 μl of serum using a miRNeasy Serum/Plasma kit (Qiagen, Hilden, Germany) according to the manufacturer’s instructions. *C*. *elegans* miR-39 miR mimic spike-in control (3.5 μl (1.6 × 108 copies/μl); Qiagen) was added before the purification of total RNA. The miScript II RT Kit (Qiagen) was used for cDNA synthesis.

#### Quantitative real-time polymerase chain reaction

The serum levels of miR-1246 (Hs_miR-1246_2 miScript Primer Assay; Qiagen) were analyzed by a quantitative real-time polymerase chain reaction (PCR) (miScript SYBR Green PCR Kit; Qiagen, Hilden, Germany) and normalized to Ce-miR-39 (Ce_miR-39_1 miScript Primer Assay; Qiagen) using a 7300 Real-Time PCR system (Applied Biosystems) according to the manufacturer’s protocol. A Mann-Whitney U-test and receiver operating characteristic (ROC) analysis were performed to analyze the difference in miR-1246 expression between ESCC patients and healthy controls.

### Radiogenomics

#### Computed tomography

All CTs were performed using a 128-detector row computed tomography (CT) system (SOMATOM Definition Flash; Siemens; Erlangen, Germany). The following imaging parameters were applied: tube voltage, 120 kVp; tube current, 160 mAs; beam pitch, 0.6; resolution 0.68 × 0.68 × 5 mm. Contrast agent (iomeprol, Iomeron 350; Eisai, Tokyo, Japan; 100 mL) was administered from the superficial vein of the upper extremity using double-head power injector (injection rate, 4.5 mL/sec). The contrast agent was followed by normal saline (30 mL) at the same injection rate. Imaging slices from the skull base to the lower were acquired 40 s after contrast agent injection.

#### Volume of interest delineation

**Figure 4:**
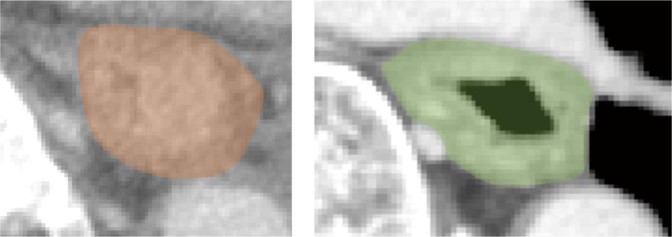
Two examples of volume of interest (VOI) delineation. In addition to the tumor, the wall and lumen of the esophagus on the slice visualizing the tumor were also included in the VOI.

A board-certified radiologist and surgeon (13 and 20 years of experience in gastrointestinal imaging, respectively) reviewed the CT, ^18^F-fluorodeoxyglucose-positron emission tomography (FDG-PET), and endoscopic images, and delineated a whole tumor volume of interest (VOI) on CT images with consensus. VOI delineation was performed with Slicer ver. 4.8.1 (https://slicer.org/). In order to avoid variation of recognition of tumor extension as much as possible, the VOIs covered the wall and lumen of the esophagus on the slice that included the ESCC (Fig. 4).

#### Feature extraction

Absolute intensity rescaling methods (−1000 to 1000 Hounsfield unit) were applied using LIFEx (https://www.lifexsoft.org). In rescaling, the upper and lower limits were set in advance and the pixel values were resampled into 64 levels. The upper limit was set to 1000 Hounsfield units and the lower limit was set to −1000 Hounsfield units; numerical values outside the range were truncated. A total of 45 features (morphology, 4; histogram, 9; texture, 32) were extracted from the VOI of each sequence using the LIFEx open source software program (Supplementary Table 1)^38^.

#### Machine learning

A Pearson’s correlation test was performed to analyze the correlation between the miR-1246 expression and each imaging feature, in order to select correlated features. Imaging features with P values of <0.05 were selected. To construct a predictive model for the miR-1246 expression, a linear regression analysis was performed using the features selected in the correlation analysis. 10-fold cross-validation was performed to calibrate generalization ability of the model. The relationship between the real and predicted miR-1246 expression levels (miR-1246_real_ and miR-1246_pred_) was shown in scatter plots and Pearson’s correlation coefficients were calculated.

#### Threshold setting to distinguish the high and low expression of miR-1246, and the Survival analysis

The threshold to distinguish the high and low expression of real miR-1246 was defined by an receiver operating characteristic (ROC) analysis of the 92 ESCC patients (survivors and non-survivors). The threshold was at the at the point of the maximum Youden index (sensitivity + specificity − 1). The survival curves of the two groups with serum miR-1246 expression levels above and below the threshold were compared using a log-rank test.

Next, the high and low miR-1246_pred_ expression groups were compared using a log-rank test. The threshold dividing the groups was same as the value calculated from miR-1246_real_. To evaluate the power of miR-1246_pred_ as a prognostic factor, univariate and multivariate Cox regression analyses were performed. In the univariate analyses, clinical factors, including age, sex, tumor staging and tumor markers, miR-1246_real_ and miR-1246_pred_ were individually analyzed. Factors identified as significant in the univariate analyses were included in the multivariate analysis. miR-1246_real_ and miR-1246_pred_ were handled separately because the purpose of the present study was to evaluate whether the prognostic ability of miR-1246_pred_ is equivalent to miR-1246_real_. Statistics and machine learning procedures were all performed using the R software program (version 3.5.1, R Foundation for Statistical Computing, Vienna, Austria).

## Supplementary information

Supplementary information.
