# Supplementary material for: Radiogenomics predicts the expression of microRNA-1246 in the serum of esophageal cancer patients
Source: Sci Rep. 2020 Feb 13;10:2532. doi: 10.1038/s41598-020-59500-7 (PMC7018689; doi:10.1038/s41598-020-59500-7)
Supplement: Supplementary file 1 — Supplementary information. [file 41598_2020_59500_MOESM1_ESM.docx]

| **Supplementary Table 1 Extracted imaging features** | | | | | |
| --- | --- | --- | --- | --- | --- |
| No | Imaging features | Type | No | Imaging features | Type |
| 1 | Mean | Histogram | 24 | GLRLM_HGRE | Texture |
| 2 | Standard deviation | Histogram | 25 | GLRLM_SRLGE | Texture |
| 3 | Minimum | Histogram | 26 | GLRLM_SRHGE | Texture |
| 4 | Median | Histogram | 27 | GLRLM_LRLGE | Texture |
| 5 | Maximum | Histogram | 28 | GLRLM_LRHGE | Texture |
| 6 | HISTO_Skewness | Histogram | 29 | GLRLM_GLNU | Texture |
| 7 | HISTO_Kurtosis | Histogram | 30 | GLRLM_RLNU | Texture |
| 8 | HISTO_Entropy | Histogram | 31 | GLRLM_RP | Texture |
| 9 | HISTO_Uniformity | Morphology | 32 | NGLDM_Coarseness | Texture |
| 10 | SHAPE_Volume (mL) | Morphology | 33 | NGLDM_Contrast | Texture |
| 11 | SHAPE_Volume (voxels) | Morphology | 34 | NGLDM_Busyness | Texture |
| 12 | SHAPE_Sphericity | Morphology | 35 | GLZLM_SZE | Texture |
| 13 | SHAPE_Compacity | Morphology | 36 | GLZLM_LZE | Texture |
| 14 | GLCM_Homogeneity | Texture | 37 | GLZLM_LGZE | Texture |
| 15 | GLCM_Energy | Texture | 38 | GLZLM_HGZE | Texture |
| 16 | GLCM_Contrast | Texture | 39 | GLZLM_SZLGE | Texture |
| 17 | GLCM_Correlation | Texture | 40 | GLZLM_SZHGE | Texture |
| 18 | GLCM_Entropy_log10 | Texture | 41 | GLZLM_LZLGE | Texture |
| 19 | GLCM_Entropy_log2 | Texture | 42 | GLZLM_LZHGE | Texture |
| 20 | GLCM_Dissimilarity | Texture | 43 | GLZLM_GLNU | Texture |
| 21 | GLRLM_SRE | Texture | 44 | GLZLM_ZLNU | Texture |
| 22 | GLRLM_LRE | Texture | 45 | GLZLM_ZP | Texture |
| 23 | GLRLM_LGRE | Texture |  |  |  |
